# Vasoactive Intestinal Polypeptide Promotes Intestinal Barrier Homeostasis and Protection Against Colitis in Mice

**DOI:** 10.1371/journal.pone.0125225

**Published:** 2015-05-01

**Authors:** Xiujuan Wu, Victoria S. Conlin, Vijay Morampudi, Natasha R. Ryz, Yasmin Nasser, Ganive Bhinder, Kirk S. Bergstrom, Hong B. Yu, Chris C. M. Waterhouse, Allison M. J. Buchan, Oana E. Popescu, William T. Gibson, James A. Waschek, Bruce A. Vallance, Kevan Jacobson

**Affiliations:** 1 Department of Pediatrics, Division of Gastroenterology, BC Children’s Hospital and the University of British Columbia, Vancouver, British Columbia, Canada; 2 Child and Family Research Institute, BC Children’s Hospital and the University of British Columbia, Vancouver, British Columbia, Canada; 3 Oklahoma Medical Research Foundation (OMRF), Oklahoma City, Oklahoma, United States of America; 4 Department of Pediatrics, University of Calgary, Calgary, Alberta, Canada; 5 Faculty of Medicine, University of Toronto, Ontario, Canada; 6 Department of Pathology, BC Children’s Hospital and the University of British Columbia, Vancouver, British Columbia, Canada; 7 Child and Family Research Institute, BC Children’s Hospital and the University of British Columbia, Vancouver, British Columbia, Canada; 8 Department of Medical Genetics, University of British Columbia, Vancouver, British Columbia, Canada; 9 Semel Institute for Neuroscience and Human Behavior, Department of Psychiatry and Biobehavioral Sciences, David Geffen School of Medicine, University of California Los Angeles, Los Angeles, United States of America; Cincinnati Children's Hospital Medical Center, University of Cincinnati College of Medicine, UNITED STATES

## Abstract

Inflammatory bowel disease is a chronic gastrointestinal inflammatory disorder associated with changes in neuropeptide expression and function, including vasoactive intestinal peptide (VIP). VIP regulates intestinal vasomotor and secretomotor function and motility; however, VIP’s role in development and maintenance of colonic epithelial barrier homeostasis is unclear. Using VIP deficient (VIPKO) mice, we investigated VIP’s role in epithelial barrier homeostasis, and susceptibility to colitis. Colonic crypt morphology and epithelial barrier homeostasis were assessed in wildtype (WT) and VIPKO mice, at baseline. Colitic responses were evaluated following dinitrobenzene sulfonic acid (DNBS) or dextran-sodium sulfate (DSS) exposure. Mice were also treated with exogenous VIP. At baseline, VIPKO mice exhibited distorted colonic crypts, defects in epithelial cell proliferation and migration, increased apoptosis, and altered permeability. VIPKO mice also displayed reduced goblet cell numbers, and reduced expression of secreted goblet cell factors mucin 2 and trefoil factor 3. These changes were associated with reduced expression of caudal type homeobox 2 (Cdx2), a master regulator of intestinal function and homeostasis. DNBS and DSS-induced colitis were more severe in VIPKO than WT mice. VIP treatment rescued the phenotype, protecting VIPKO mice against DSS colitis, with results comparable to WT mice. In conclusion, VIP plays a crucial role in the development and maintenance of colonic epithelial barrier integrity under physiological conditions and promotes epithelial repair and homeostasis during colitis.

## Introduction

The intestinal epithelium and overlying secreted mucus layer are all that separates the host from its intestinal luminal environment. Impaired intestinal epithelial barrier integrity has been shown to increase susceptibility to immune-mediated inflammatory disorders, including inflammatory bowel disease (IBD) [1]. While there are many factors controlling intestinal barrier function, the enteric nervous system (ENS) plays a critical, yet incompletely understood role in regulating this key aspect of gut health. The ENS regulates gastrointestinal (GI) physiology and function, in part through secretion of neuropeptides, including VIP [2]. In IBD, intestinal inflammation can disrupt ENS structure and function, causing patients to experience abdominal pain, urgency and diarrhea, even during quiescent disease [2]. Overt intestinal inflammation has been associated with significant decreases in VIP+ neurons as well as altered expression of VIP+ neuronal subpopulations [3, 4]. Furthermore, it was recently shown that VIP and its receptor VPAC1 could not be detected in tissues from IBD patients suffering severe mucosal damage [5]. These findings suggest dysregulated VIP responses may contribute to IBD pathogenesis, but at present, the role of VIP in maintaining intestinal health is largely unexplored.

VIP fibers form a dense neural network throughout the lamina propria that likely innervate intestinal epithelial cells (IEC) [6]. Aside from neurons, immune cells including T cells, B cells, mast cells, and eosinophils produce VIP [7]. VIP activates G-protein coupled receptors VPAC1 and VPAC2, which are abundantly expressed throughout the gut [6]. However, the role of VIP and its receptors during colitis remains unclear. VIP treatment has been shown to reduce the severity of 2,4,6-trinitrobenzene sulfonic acid (TNBS)-induced colitis [8, 9] and to protect IEC barrier integrity during *Citrobacter rodentium*-induced colitis [10]. However, higher concentrations of VIP can lead to worsening of TNBS-colitis [8] as well as impaired barrier function, at least *in vitro* [10]. Moreover, variable responses have been associated with DSS challenge with exacerbated disease in mice lacking VPAC2 compared to wildtype (WT) mice, whereas *Vpac1 -/-* mice [11] and *Vip* ^*-/-*^ (hereafter referred to as VIPKO) mice had milder disease than WT mice, and use of pharmacological inhibition of VIP receptors in WT mice was also associated with milder disease [12, 13]. VIP KO mice also developed a milder clinical response to TNBS-induced colitis than WT mice, although histological scores and cytokine levels in the colon did not differ between the two strains of mice and splenocytes from TNBS-treated VIP KO mice exhibited an enhanced proliferative response to anti-CD3/CD28 stimulation in *vitro* [14]. Despite these conflicting results, VIP is undoubtedly an important regulator of normal gut function and further characterization of its actions are required under both physiological and pathological conditions.

In this study, using VIPKO mice we show that the absence of VIP leads to abnormal colonic crypt morphology and function, reflecting baseline defects in IEC proliferation and migration. VIPKO mice also suffered impaired goblet cell development, leading to significantly reduced expression of mucin 2 (Muc2) and trefoil factor 3 (Tff3) as well as overt intestinal barrier dysfunction. These changes were associated with reduced expression of Cdx2, a transcription factor known to modulate cell proliferation, migration and differentiation [15]. Notably VIPKO mice also showed heightened susceptibility to DNBS and DSS-induced colitis, while treatment with VIP rescued the phenotype, protecting VIPKO mice against DSS-colitis. Our data thus identifies VIP as an important regulator of IEC homeostasis and function; therefore alterations in its expression or function may well contribute to the symptomatology and pathogenesis of IBD.

## Methods

### Mice and epithelial cell lines

Eight-week-old VIPKO mice (C57BL/6 background) [16] were bred at the Child and Family Research Institute animal facility. Male mice were used in all experiments due to the consistency seen in their DSS and DNBS-induced colitis. As no significant differences were found between littermates (*Vip* ^+/+^) and C57BL/6 mice from Charles River under physiological and pathological conditions, age matched male C57BL/6 mice were purchased from Charles River Laboratories (St. Constant, QC, Canada) and housed as previously described [17]. All mice were fed a standard chow diet (LabDiets, Picolab rodent diet 5053) with an n-6: n3 polyunsaturated fatty acid (PUFA) ratio of 8:1. The protocols were approved by the University of British Columbia’s Animal Care Committee and in direct accordance with guidelines of the Canadian Council on the Use of Laboratory Animals. Human Caco2 (ATCC HTB-37) and HT29 (ATCC HTB-38) IEC lines were cultured in Dulbecco’s modified Eagle’s medium supplemented with 10% fetal bovine serum, 20mM HEPES, 1% glutamine, antibiotics penicillin (100U/ml) and streptomycin (100μg/ml) (Sigma Chemicals Co., St. Louis, MD). Cells were maintained at 37°C in a humidified incubator of 5% CO_2_.

### Induction of DNBS colitis in vivo and clinical scoring ex vivo

Colitis was induced by intra-rectal injection of 6 mg of DNBS in 100μL 50% ethanol via a polyethylene catheter (PE-50) as previously described [18]. Following euthanization, colonic (distal colon) tissues were assessed for macroscopic damage [sum of the following scores: extent of tissue adhesion (0–2), colon wall thickness (in mm), macroscopic ulceration (0–10), fat wrapping (0–5)] as well as histological damage [sum of the following scores: inflammatory cell infiltrate (0-absent, 3-transmural), loss of epithelial architecture (0-normal, 3-severe), presence of crypt abscesses (0–1), and goblet cell depletion (0–3), and tissue thickness at 100X magnification (0-normal, 1–50% increase, 2–100% increase, 3->100% increase)] [18]. Luminal stool contents were collected from the colon once opened, weighed, and then left to dry at 37°C for 48 hrs. The contents were weighed again, with the water content defined as the percentage of the initial stool weight lost after drying.

### Induction of DSS colitis, clinical scoring and VIP treatment in vivo

A modified version of the protocol described by Stillie R *et al* was used [19]. Briefly, acute colitis was induced by adding DSS (36,000–55,000 kDa, MP Biomedicals #160110, Solon, Ohio, USA) to sterile drinking water at a concentration of 3% (w/v) for 7 days, followed by a switch to tap water for 3 days. The first day of DSS feeding was defined as day 0, and the mice were given DSS until day 7 followed by 3 days of water. Control mice were fed with regular water (without DSS). Mice were monitored for weight loss, stool consistency, rectal bleeding (hemoccult strips, Beckman Coulter) and severity of colitis as previously described [20]. For VIP treatment, 6–8 weeks old mice were treated with 0.5 nmol VIP in saline administered by intraperitoneal (ip) injection daily from day 0 to day 9 after DSS treatment. The dose chosen was in keeping with doses used previously in murine models of colitis [10]. Control mice received saline alone by ip. injection. Similarly, VIP was given to naïve mice that were not exposed to DSS treatment for 10 days. Mice were euthanized on day 10 and the whole colon removed for evaluation of macroscopic damage, and a section of the distal colon was then evaluated for histological damage and the water content in the stool was quantified.

### Histopathological scoring

Histological damage scores were calculated as previously described [20]. Briefly, distal colonic segments (0.5cm) were fixed with 10% formalin and embedded in paraffin. Cross sections of the colon (5μm) were cut and mounted on slides. Tissue sections were stained with hematoxylin and eosin (H&E) and cell morphology was viewed using light microscopy. Six tissue sections from each animal were coded and examined by two blinded observers to prevent observer bias. Tissue sections were assessed (each separated by at least 500μm) under a Nikon Eclipse 400 light microscope and averaged to obtain a mean histological damage score. The following criteria was used for scoring- inflammation (0–3), transmural inflammation (0–3) and crypt damage (0–4). The score of each feature was multiplied by a factor (1–4) according to the percentage of epithelial involvement. The maximum damage score with this system is 40.

### Measurement of Intestinal Permeability

The FITC-dextran assay was performed as previously described [21]. Briefly, mice were given 0.1 ml of 80 mg/mL FITC-dextran (Sigma; FD4) in PBS by enema 2h prior to sacrifice. Blood was collected by cardiac puncture and added to 3% acid-citrate dextrose; plasma was collected and fluorescence was measured using a Wallac Victor fluorimeter (Perkin-Elmer Life Sciences, Boston, MA).

### RNA extraction and quantitative real-time polymerase chain reaction (PCR)

Total RNA was extracted from freshly isolated colonic tissues as well as HT-29-Cl.16E cells treated with recombinant VIP (0.6μM, 1μM and 3μM) by using Qiagen RNeasy plus mini kit. RNA was reverse-transcribed using Superscript II reverse transcriptase (Invitrogen) and qPCR and quantification was carried out as previously described [17]. Quantitative PCR was carried out on a Bio-Rad MJ Mini-Opticon Real-Time PCR System (Bio-Rad), using IQ SYBR Green Supermix (Bio-Rad). Individual primer sequences are listed in Supplementary Information (S1 Table). After completion of the cycling process, samples were subjected to a temperature ramp (from 53 to 95°C) with continuous fluorescence monitoring for melting curve analysis. For each PCR product, a single narrow peak was obtained by melting curve analysis at the specific melting temperature, indicating specific amplifications. Primer pair efficiency was tested according to manufacturer's instructions. Quantification was carried out with Gene Ex Macro OM 3.0 software (Bio-Rad) where PCR efficiencies for each of the primer sets were incorporated into the final calculation. The ΔΔCt method was used to calculate the relative amount of specific RNA present in a sample, from which the fold induction of transcription of the gene was estimated by comparison to values relative to the control samples. Data are expressed as means ± SD.

### Epithelial cell apoptosis

The terminal deoxynucleotidyl transferase-mediated dUDP nick-end labeling (TUNEL) staining was employed using the *in situ* cell death detection kit (Roche Diagnostics, Mannheim, Germany) according to manufacturer's instructions, as previously described [17]. Briefly, colonic tissue sections of 4μm thickness were mounted on glass slides, deparaffinized, hydrated and treated for 15 min with proteinase K (50 μg/ml). After rinsing, the TUNEL reaction mixture was added to the samples. The slides were incubated in a humidified chamber for 60 min at 37°C in the dark. Slides were counterstained with 4,6-diamidino-2-phenylindole (DAPI). For each section, the average number of TUNEL +ve cells per visual field was calculated at 400x magnification.

### Immunohistochemistry

Immunofluorescence staining was performed and assessed as described previously [17]. Primary antibodies included rabbit monoclonal anti-ki67 (Thermo scientific), rabbit polyclonal antisera against murine colonic mucin Muc2 (1:50; a gift from Jan Dekker), rabbit polyclonal antisera against TFF3 (1:200; a gift from D. Podolsky), polyclonal rabbit anti-VIP (Immunostar), goat polyclonal anti-carbonic anhydrase (CA) I (Santa Cruz), rat anti-BrdU monoclonal antibody (1:200, AbD Serotec) and rabbit anti-serotonin (5HT) (Antibodies incorporated). For immunohistochemistry of longitudinal muscle myenteric plexus preparations, colon tissues were prepared as described previously [22]; primary antibodies included polyclonal rabbit anti-VIP (1:1000; Immunostar), rabbit anti-neuronal nitric oxide synthase (NOS—1:500; Transduction), rabbit anti-substance P (SP—1:1000; Immunonuclear Corp), and goat anti-neuropeptide Y (NPY—1:1000; a gift from Thue Schwartz).

### 5-Bromo-2-deoxyuridine (BrdU) incorporation

Mice were administrated by i.p. injection with 10 mg ml^-1^ of BrdU (Sigma) as described previously [23]. The colons were excised at 72h post-injection. Distal colon segments were fixed as described above. Following immunostaining for BrdU, the number of BrdU+ve IEC per crypt was quantified, using only intact, well-oriented crypts.

### Western blot analysis

Monolayers were lysed using 300 μl of lysis buffer [150mM NaCl, 20mM Tris pH 7.5, 1mM EDTA, 2.5mM sodium pyrophosphate, 1mM β-glycerophosphate, 1mM phenylmethylsulfonyl fluoride (PMSF), 1mM sodium orthovanadate, and 1mM sodium fluoride, with 1% Triton X-100, 1% phosphatase inhibitor cocktail (Thermo Scientific) and protease inhibitor cocktail tablets (Roche, Mannheim, Germany)]. Lysates were resolved by 15% sodium dodecyl sulfate-polyacrylamide gel electrophoresis (SDS-PAGE) and then transferred onto 0.2 μm PVDF membranes (Bio-Rad). Rabbit anti-cyclin D1 and β-actin antibodies were all used at 1:1000 dilutions and secondary anti-rabbit-HRP labelled antibodies were used at 1:2000. Blots were visualized by an enhanced chemiluminescence detection system (Perkin Elmer). Incubation with anti-β-actin was used as a loading control. The intensity of bands was quantified with Image J software and the ratio of cyclin D1 intensity divided by β-actin intensity was normalized against the control.

### Goblet cell enumeration

Periodic acid-Schiff (PAS) staining was carried out as described previously [24]. Under baseline condition, the number of mature goblet cells was expressed as the total number of PAS+ve cells per 100 epithelial cells. Phenotypically mature goblet cells were assessed based on the intensity of staining, the size of the apical region, their location on the crypt base-to-surface axis, and morphology [24]. Under DSS-induced colitis condition, the results were expressed as total number of PAS+ve cells per high power field (HPF, 200X).

### Statistical analysis

Analyses were conducted with Graph Pad Prism 5 statistical software for Windows (GraphPad Software, San Diego, California USA). Results are expressed as mean value with standard error of the mean (SEM). Differences between means were calculated by either one or two-way analysis of variance (ANOVA), or *t*-tests where appropriate. Specific differences were tested with the Student-Newman-Keuls test where *P* of < 0.05 was considered statistically significant.

## Results

### VIP deficient mice display aberrant crypt structure at baseline

**Fig 1:**
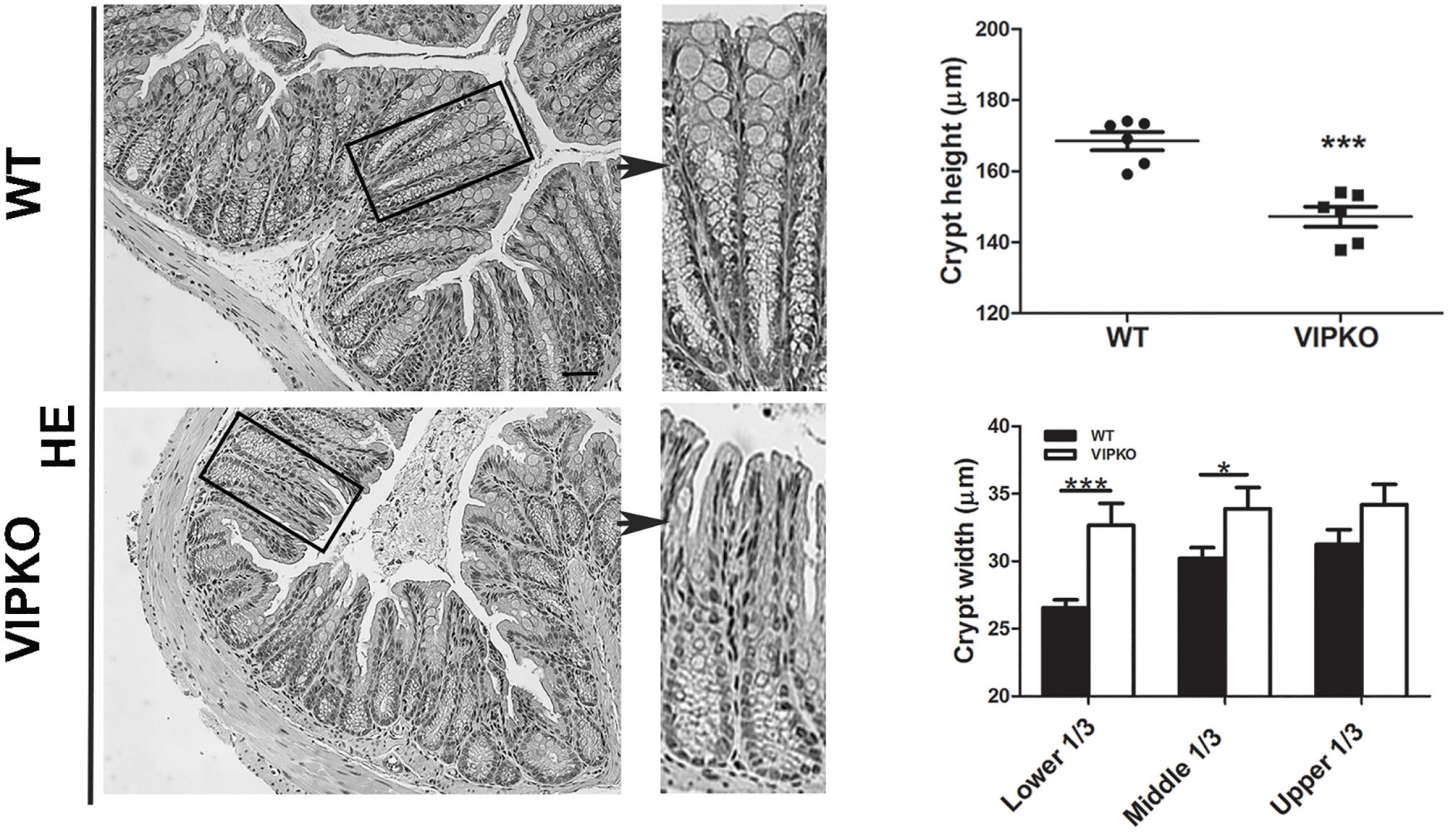
VIPKO mice display aberrant crypt structure at baseline. Representative H&E stained colon sections and quantitative analysis of crypt height and width in WT and VIPKO mice. n = 6–10 animals/ group, results are represented as means ± SEM, *P<0.05, ***P<0.001.

To investigate the role of VIP in colonic crypt morphogenesis and barrier homeostasis, VIPKO mice were examined under baseline conditions. We first assessed colonic tissues for the presence and location of VIP. In WT mice, VIP was distributed throughout the neural networks, in the lamina propria, submucosa (S1A Fig) and myenteric plexus, while it was absent in VIPKO mice (S1B Fig). No gross differences in SP, NPY or NOS immunoreactivity were observed in the myenteric plexus when comparing VIPKO and WT mice (data not shown), confirming a VIP restricted deficiency. Deletion of the VIP gene was confirmed by PCR (S1C Fig). Expression of VIP receptors, VPAC1 and VPAC2, was similar between WT and VIPKO mice (Table 1). Histologically, VIPKO mice displayed abnormal colonic crypt morphology compared to WT mice with significantly shorter (147.21 ± 2.81 μm *vs*. 168.47 ± 2.58 μm, *p*<0.001, Fig 1) and wider crypts.

**Table 1:** Real time PCR analysis of genes in wild type and VIPKO mice.

Gene	WT mice	VIPKO mice	*p value*
*Vpac1*	1.07 ± 0.15	1.63 ± 0.21	0.11
*Vpac2*	1.09 ± 0.17	0.98 ± 0.11	0.58
*Notch1*	1.03 ± 0.10	1.26 ± 0.14	0.21
*Hes1*	1.04 ± 0.12	0.77 ± 0.13	0.16
*Math1*	1.01 ± 0.08	0.83 ± 0.09	0.24
*Klf4*	1.02 ± 0.09	0.96 ± 0.12	0.71
*Klf5*	1.04 ± 0.12	1.19 ± 0.14	0.42
*Wif1*	1.01 ± 0.07	1.00 ± 0.09	0.93
*Relmβ*	1.27 ± 0.38	2.63 ± 0.69	0.09

### VIP enhances colonic crypt function at baseline

**Fig 2:**
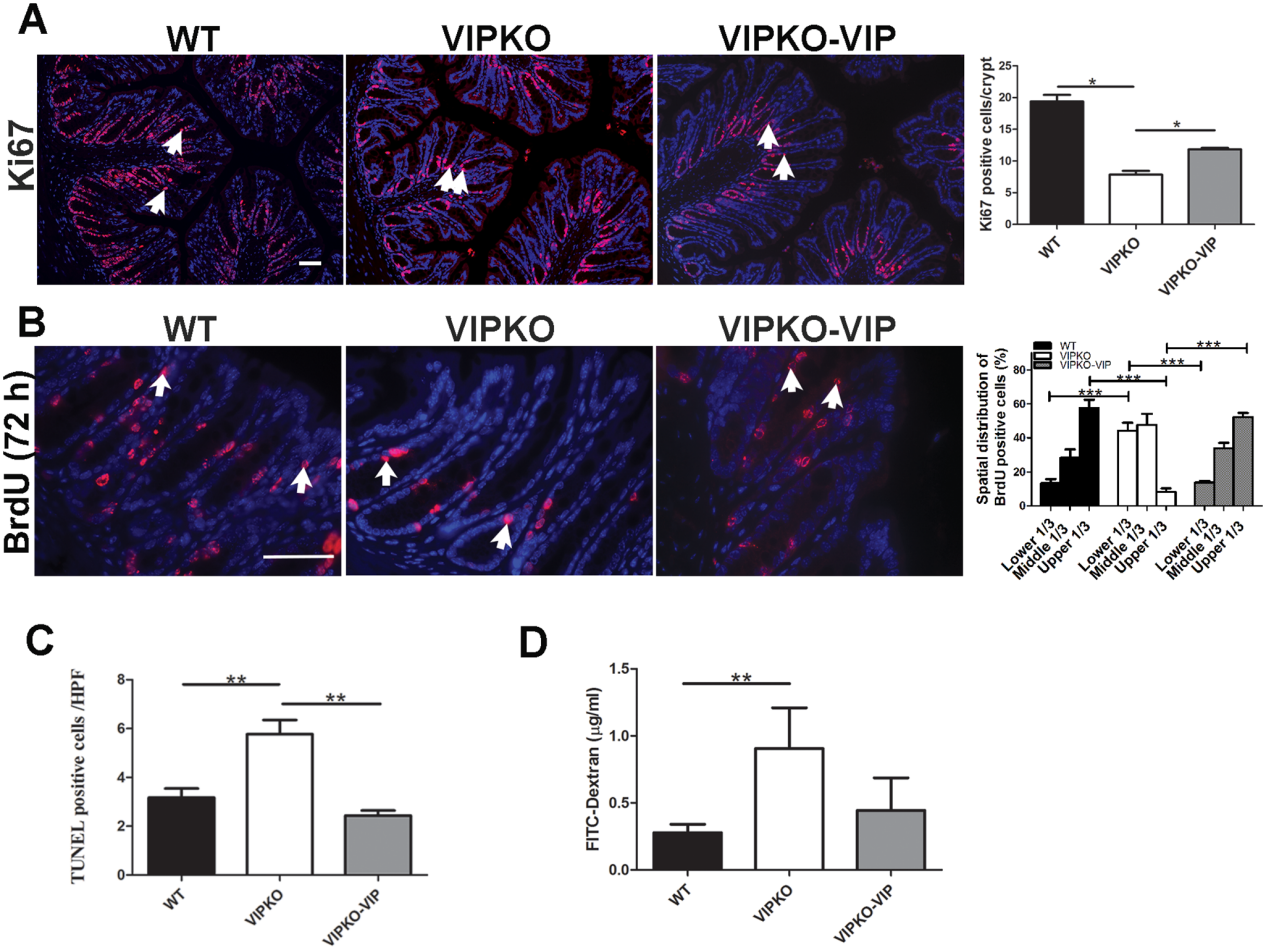
VIP enhances colonic crypt function at baseline. Exogenous VIP was administered to VIPKO mice (VIPKO-VIP) daily for 10 days. Immunostaining for Ki67 and quantitative analysis of Ki67+ve cells (A). Immunostaining for BrdU+ve cells and calculated spatial distribution at 72h post injection (B). Quantitative analysis of TUNEL +ve crypt IEC (C). Epithelial permeability measured by FITC dextran (D); n = 6–10 animals/group, results are represented as means ± SEM, *P<0.05, **P< 0.01, ***P<0.001. Scale bar = 50 μm.

To assess colonic crypt cellular dynamics, IEC proliferation, migration, apoptosis, and barrier integrity were examined. The proliferating colonic crypt cell population, marked by Ki67 (non-G_0_ cycling cells) and BrdU (S-phase) was significantly reduced in VIPKO mice compared to WT mice (7.85 ± 0.60 *vs*. 19.39 ± 0.68, p<0.05, Fig 2A) and (3.71 ± 0.20/crypt *vs*. 5.37 ± 0.22/crypt, p<0.01, Fig 2B) respectively. Furthermore, the number of TUNEL +ve IEC was significantly higher in VIPKO mice (5.77 ± 0.57/HPF *vs*. 3.17 ± 0.37/HPF, p<0.01, Fig 2C) suggesting that an altered balance between cell proliferation and cell death might be responsible for the aberrant crypt morphology. Further evaluation of IEC migration dynamics using 72h post-BrdU labeling [23] showed significantly lower BrdU+ve cell migration rates in VIPKO mice; with BrdU+ve cells distributed throughout the crypts in WT mice, whereas positive cells were located predominantly in the lower half of VIPKO crypts (Fig 2B). To further evaluate cell migration, colonic crypts were divided into lower, middle, and upper sections. Compared to WT mice, BrdU+ve cells in VIPKO mice were predominantly in lower and middle crypt sections (91.7% *vs*. 42.6%, p<0.001) with dramatically fewer BrdU+ve cells in upper sections (8.3% *vs*. 57.6%, p<0.001) compared to WT mice (Fig 2B), supporting a role for VIP in promoting IEC migration and homeostasis. To determine whether VIP impacts on intestinal barrier integrity *in vivo*, FITC-dextran was given via enema. VIPKO mice showed significantly higher serum levels of the FD4 probe, compared to WT mice (0.90 ± 0.29 μg mL^-1^ *vs*. 0.28 ± 0.06 μg mL^-1^, respectively, *p*<0.01, Fig 2D), indicating impaired barrier integrity in VIPKO mice.

To rule out the possibility that the VIPKO intestinal phenotype was due to developmental defects during embryogenesis and/or neonatal development, the impact of VIP reconstitution was assessed. VIP administered to VIPKO mice (6–8 weeks old) significantly reduced the number of abnormal colonic crypts (80.14 ± 1.45% *vs*. 63.61 ± 7.74%, p<0.01), resulting in a colonic architecture more closely resembling WT mice than untreated VIPKO mice (data not shown). VIP treatment significantly increased Ki67+ve cell numbers in the distal colon, compared to untreated VIPKO mice (11.85 ± 0.42 *vs*. 7.85 ± 0.60, p<0.05, Fig 2A) expanding the crypt proliferative zone. Moreover, VIP treatment significantly increased crypt cell migration, significantly reducing the number of BrdU +ve epithelial cells in the lower third of crypts (13.7% ± 0.8 *vs*. 44.1% ± 4.8, p <0.01) while increasing BrdU +ve cell numbers in the upper third of crypts (52.3% ± 2.1 *vs*. 8.3% ± 2.0, p <0.001, Fig 2B), at 72h post injection, reaching levels similar to WT mice. Conversely, VIP treatment was associated with a significant reduction in number of TUNEL +ve IEC in VIPKO mice (2.43 ± 0.21 /HPF *vs*. 5.77 ± 0.57/HPF, p<0.01, Fig 2C). Lastly, VIP treatment attenuated intestinal barrier disruption in VIPKO mice, since VIP treated VIPKO mice showed lower serum levels of the FD4 probe than VIP KO mice, declining to levels close to WT mice (Fig 2D).

Taken together, these data suggest that VIP promotes colonic epithelial homeostasis under physiological conditions.

### VIP regulates colonic goblet cell numbers and function at baseline

**Fig 3:**
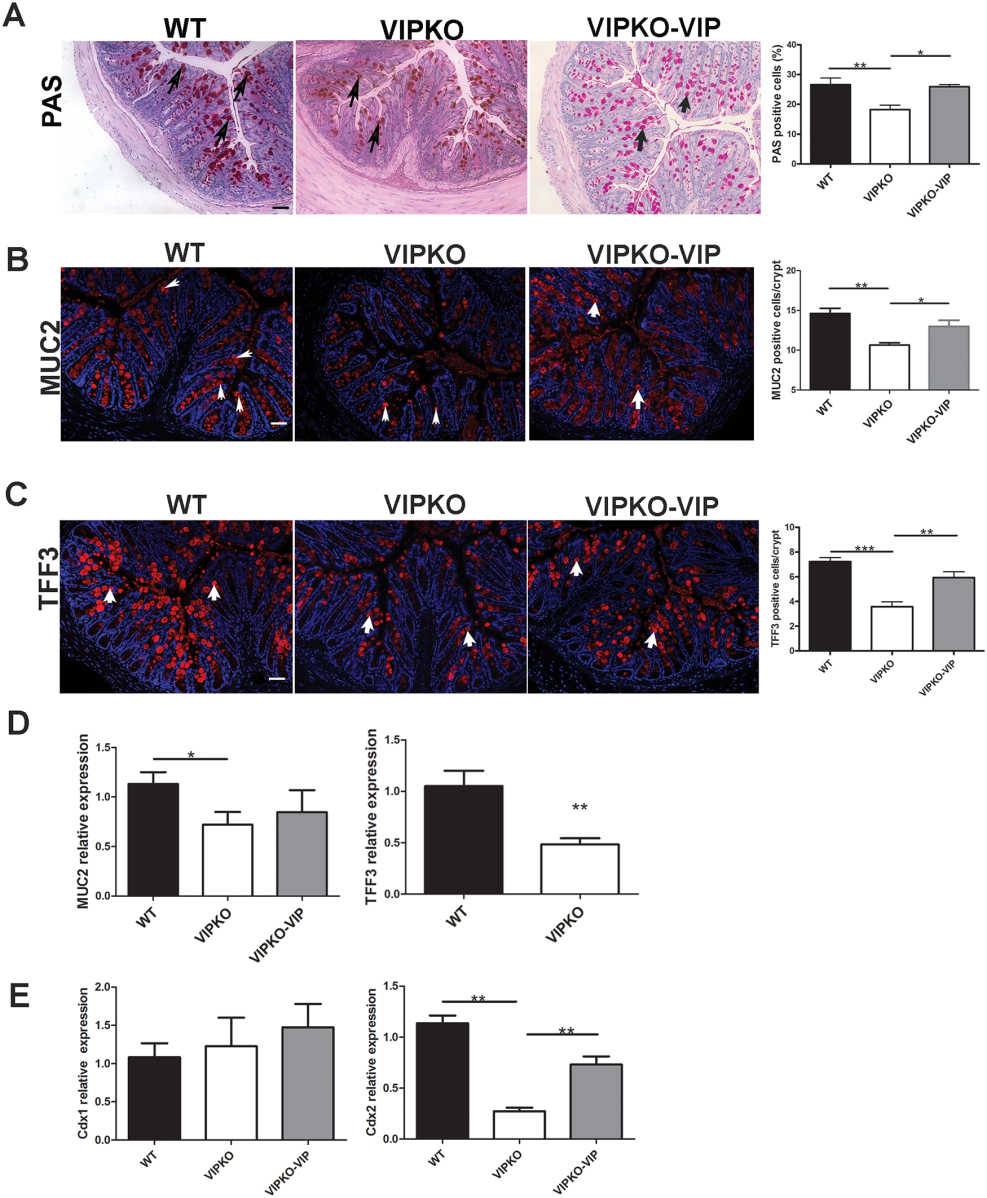
VIP regulates colonic goblet cell numbers and function at baseline. Exogenous VIP was administered to VIPKO mice (VIPKO-VIP) daily for 10 days. Selective labeling of neutral mucins with PAS and quantification of PAS+ve cells as a percentage of total IEC/crypt (A). Immunostaining and quantitative analysis of colonic Muc2+ve cells (B) and Tff3 +ve cells (C). Relative expression of colonic Muc2 and Tff3 (D), and Cdx1 and Cdx2 (E); n = 4–6 animals/group, results are represented as means ± SEM, *P<0.05, **P< 0.01, ***P<0.001. Scale bar = 50 μm. Arrows highlight cells that are positive in PAS staining (A), Muc2 staining (B) and Tff3 staining (C).

As previous studies have shown a link between VIP and goblet cell production of Muc2 and the trefoil proteins [25, 26], we next focused on colonic goblet cell distribution, maturation, and secretory capacity in naïve WT, VIPKO and VIP treated VIPKO mice. Selective labeling of neutral mucins with PAS showed numerous morphologically mature PAS+ve goblet cells distributed throughout the crypts of WT mice (Fig 3A), VIPKO mice had fewer PAS +ve cells and were comparatively lacking mature apical PAS+ve goblet cells. Enumeration of PAS+ve cells [24] confirmed that VIPKO mice had significantly fewer PAS +ve goblet cells compared to WT mice (18.22% ± 1.48 *vs*. 26.58% ± 2.23, respectively, *p*<0.01, Fig 3A). VIP treatment of VIPKO mice restored the number of PAS +ve goblet cells in VIPKO mice, suggesting the rescue of goblet cellular dynamics. To further characterize goblet cell function, colonic tissues from the aforementioned three groups of mice were examined for the location and expression of Muc2, the major secretory mucin in the intestine as well as Tff3 a bioactive peptide involved in epithelial migration and injury repair [23, 24]. VIPKO mice displayed markedly fewer Muc2 +ve goblet cells throughout their distal colon than WT mice (10.65 ± 0.23/crypt *vs*.14.62 ± 0.52/crypt, p<0.01, Fig 3B), and significantly reduced Muc2 gene transcript levels (36% decrease, p<0.05, Fig 3D). VIPKO mice also displayed markedly reduced Tff3 staining compared to WT mice, especially in the upper portions of their crypts (Fig 3C), together with significantly lower numbers of Tff3 +ve cells (3.57 ± 0.32/crypt *vs*. 7.22 ± 0.27/crypt, p<0.001, Fig 3C) and significantly lower Tff3 mRNA levels (54%, *p*<0.01, Fig 3D). VIP treatment significantly increased the number of Muc2+ve and Tff3+ve cells per crypt in VIPKO mice (12.99 ± 0.77/crypt *vs*. 10.65 ± 0.23/crypt and 5.93 ± 0.32/crypt *vs*. 3.57 ± 0.32, respectively, p<0.05, Fig 3B and 3C). Consistent with the observations of Yusta et al [13] and Lelievre et al [16] small bowel villus/ crypt length in VIPKO mice was increased as compared to WT mice (data not shown).

We next evaluated whether the defective phenotype involved all colonic epithelial cell lineages. Staining for 5-hydroxytryptophan (5-HT), an enteroendocrine cell (EEC) marker and for carbonic anhydrase-I (CA-1), a marker of mature columnar epithelial cells showed no significant differences in staining pattern or distribution between VIPKO mice and WT mice, suggesting the defect in goblet cells seen in VIPKO mice was selective (S2 Fig).

Collectively, these data suggest that VIP regulates goblet cell numbers and function under physiological conditions.

### VIP induces Cdx2 signaling

As Wnt and Notch signaling pathways play a critical role in intestinal homeostasis, we examined whether VIP deficiency altered the expression of target genes associated with these two signaling pathways. No differences in Notch1, Hes1, Math1, Krüppel-like factor 4 (KLF4), KLF5 and Wif1 mRNA expression were observed in colonic tissues of VIPKO mice compared to WT mice (Table 1) indicating these regulators of IEC progenitor fate were unaffected by VIP deficiency, at least at the transcript level [27, 28].

We next focused on the intestine specific caudal-related homeobox transcription factors, Cdx1 and Cdx2, given their strategic role in regulating intestinal homeostasis [29–31]. We observed a significant reduction in gene transcript levels of Cdx2 (but not Cdx1) in VIPKO mice compared to WT mice (0.27 ± 0.03 *vs*. 1.13 ± 0.07, p<0.01, Fig 3E), suggesting that VIP may act via Cdx2 expression. Importantly, VIP administration to VIPKO mice significantly increased Cdx2 gene expression (0.73 ± 0.08 *vs*. 0.27 ± 0.03, Fig 3E, p<0.01), but did not change Cdx1 gene expression. These data suggest that VIP induces Cdx2 signaling, but not Cdx1 signaling.

### VIP treatment of epithelial cells directly impacts their homeostasis

**Fig 4:**
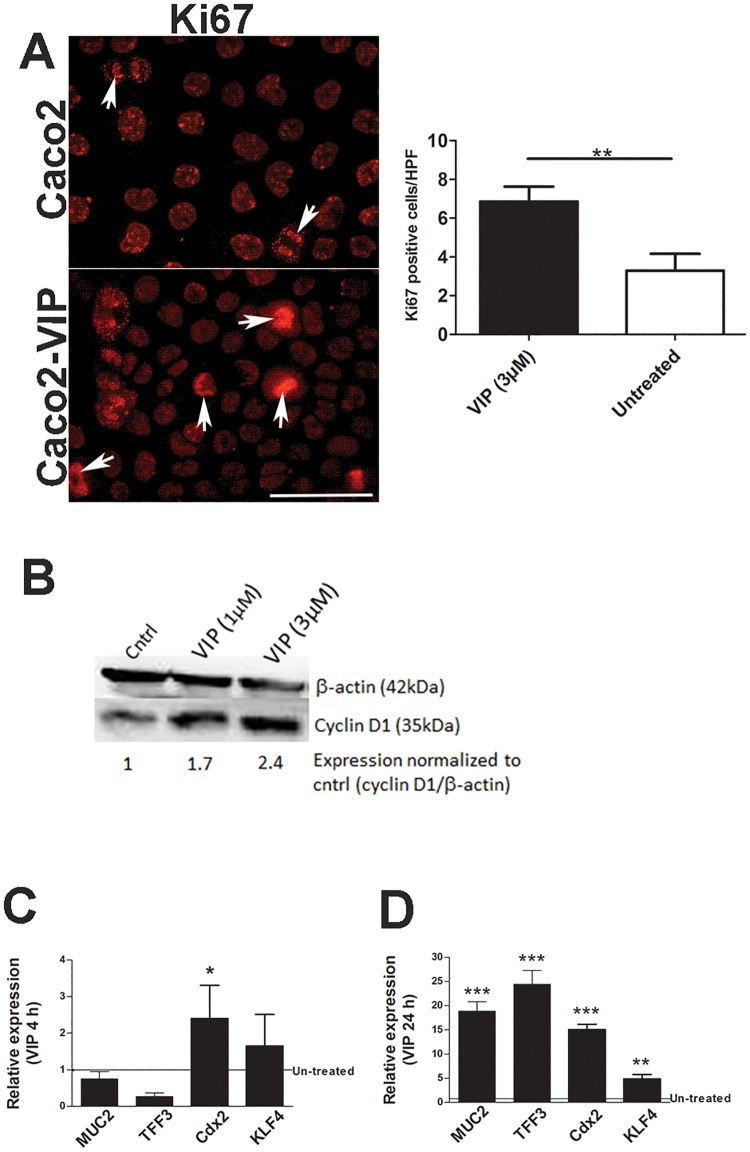
VIP treatment of IEC directly impacts epithelial homeostasis. Caco2 cells were treated with recombinant VIP (3 μM) for 24h and stained with Ki67 (A); n = 3 experiments, results are represented as means ± SEM. Caco2 cells were treated with recombinant VIP (1 μM, 3 μM) for 24h and cell lysates were analyzed for protein expression of β-actin and cyclin D1 (B). HT-29 cells were treated with recombinant VIP (3 μM) for 4h (C) and 24h (D) and analyzed for expression of MUC2, Tff3, Cdx2, and KLF4, n = 3 experiments, results are represented as means ± SEM, *P<0.05, **P< 0.01, ***P<0.001. Initial experiments, included treatment with recombinant VIP (0.6 μM, 1 μM and 3 μM), but data not included for lower doses as data most consistent with VIP (3 μM). Arrows highlight Ki67 +ve cells (A).

To clarify the impact of VIP on colonic epithelial cells, semi-confluent Caco2 epithelial cell monolayers were treated with recombinant VIP (3μM) [32, 33] for 24h and stained with Ki67. As shown in Fig 4A, and consistent with our *in vivo* data, VIP treatment significantly increased Ki67+ve cell numbers compared to untreated monolayers (p<0.01), indicating that VIP can directly induce IEC proliferation. Consistent with this result, we also found that VIP induces the expression of cyclin D1, another cell proliferation marker [34] (Fig 4B). To further explore the mechanism by which VIP regulates intestinal goblet cell homeostasis, human HT-29 cells were treated with recombinant VIP (3μM) [32, 33] for 4h and 24h and analyzed for expression of MUC2, Tff3, and Cdx2, and KLF4 (Fig 4C and 4D). VIP treatment significantly increased gene transcript levels of Cdx2 at 4h and of Tff3, Cdx2, KLF4, MUC2 (and other MUC genes, data not shown) at 24h. These data strengthen the link between VIP and Cdx2, and demonstrate that VIP can directly induce transcription of genes required for goblet cell maturation and function.

### VIP deficient mice exhibit increased susceptibility to DNBS-induced colitis

**Fig 5:**
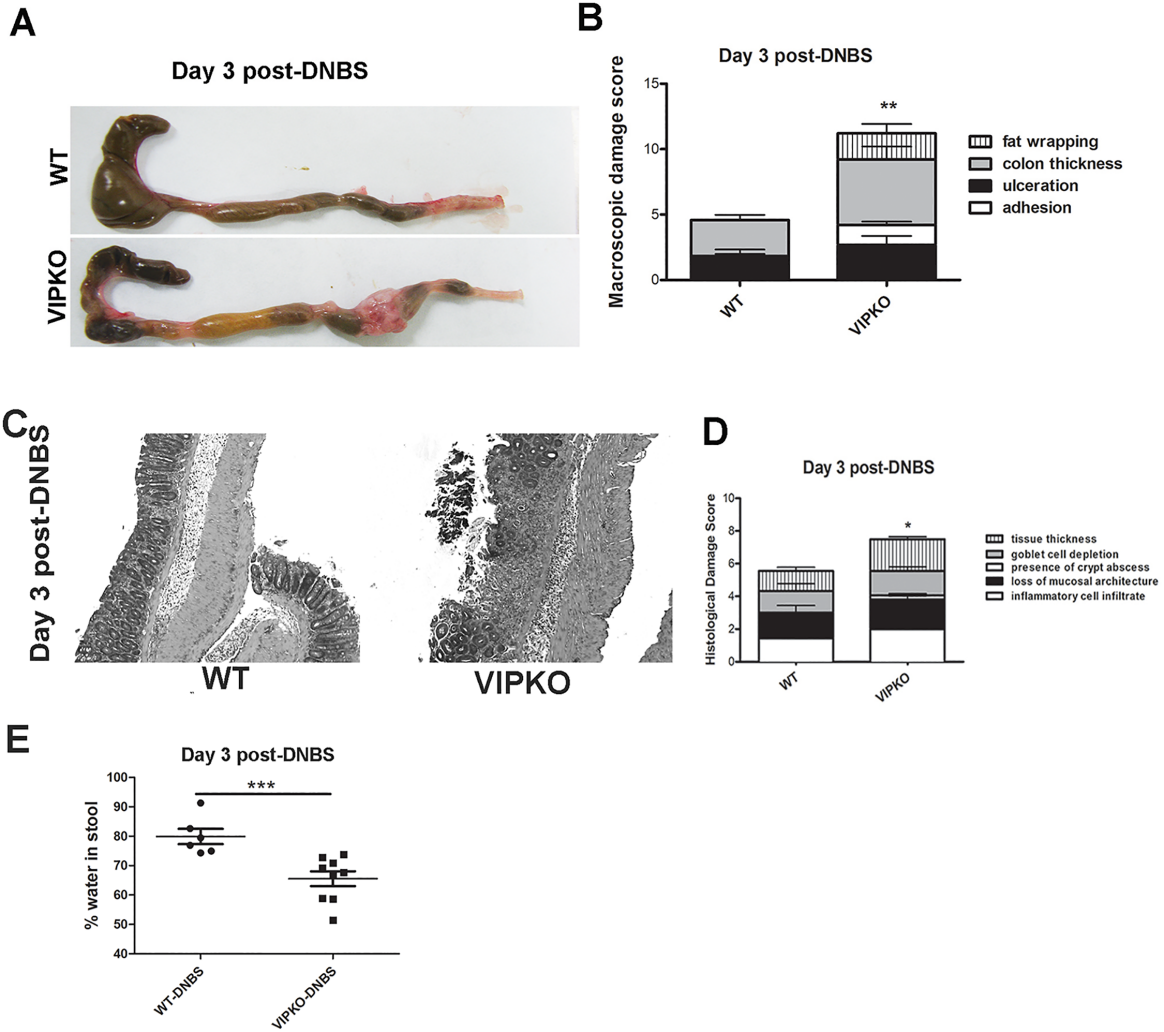
VIPKO mice exhibit increased susceptibly to chemically induced DNBS- colitis. At day 3 post-DNBS treatment VIPKO mice display shrunken ceca and significant thickening/damage of the mid to distal colon with fat wrapping when compared to WT mice (A), with significantly increased macroscopic damage scores (B). Representative H&E staining of Day 3 post-DNBS treated WT and VIPKO mice (C), when scored histologically (D) shows a significant increase in overall damage in VIPKO tissues compared to WT. Water content of luminal stool expressed as the percentage of the initial stool weight lost after drying at 37°C for 48h (E); n = 6–9 animals/group, results are represented as means ± SEM, *P<0.05, **P<0.01.

Having demonstrated an alteration in colonic IEC homeostasis and epithelial barrier integrity in VIPKO mice, under physiological conditions we next determined if VIPKO mice were more susceptible to chemically induced colitis. Mice were first challenged with DNBS. At day 3 post-DNBS, mice were euthanized and the entire large bowel of VIPKO mice showed increased damage compared to WT mice with shrunken ceca and significant mid to distal colonic thickening, often accompanied by complete fat wrapping of the corresponding segment of intestinal tissue (Fig 5A). Interestingly, fat wrapping is commonly associated with fibrostenotic resected intestinal tissue from Crohn’s disease patients. Closer assessment of macroscopic damage revealed significantly increased adhesion of tissues (p<0.05), fat wrapping (p<0.01) as well as significantly increased overall macroscopic damage scores in VIPKO mice compared to WT (p<0.01, Fig 5B). Histologically, at day 3 post-DNBS treatment, VIPKO mice exhibited significantly increased histological damage scores compared to WT (p<0.05, Fig 5C and 5D). Colonic luminal fluid as a percentage of total luminal content post-DNBS treatment was significantly lower in VIPKO mice than WT mice (p<0.05, Fig 5E).

### VIP protects mice against DSS-induced colitis

**Fig 6:**
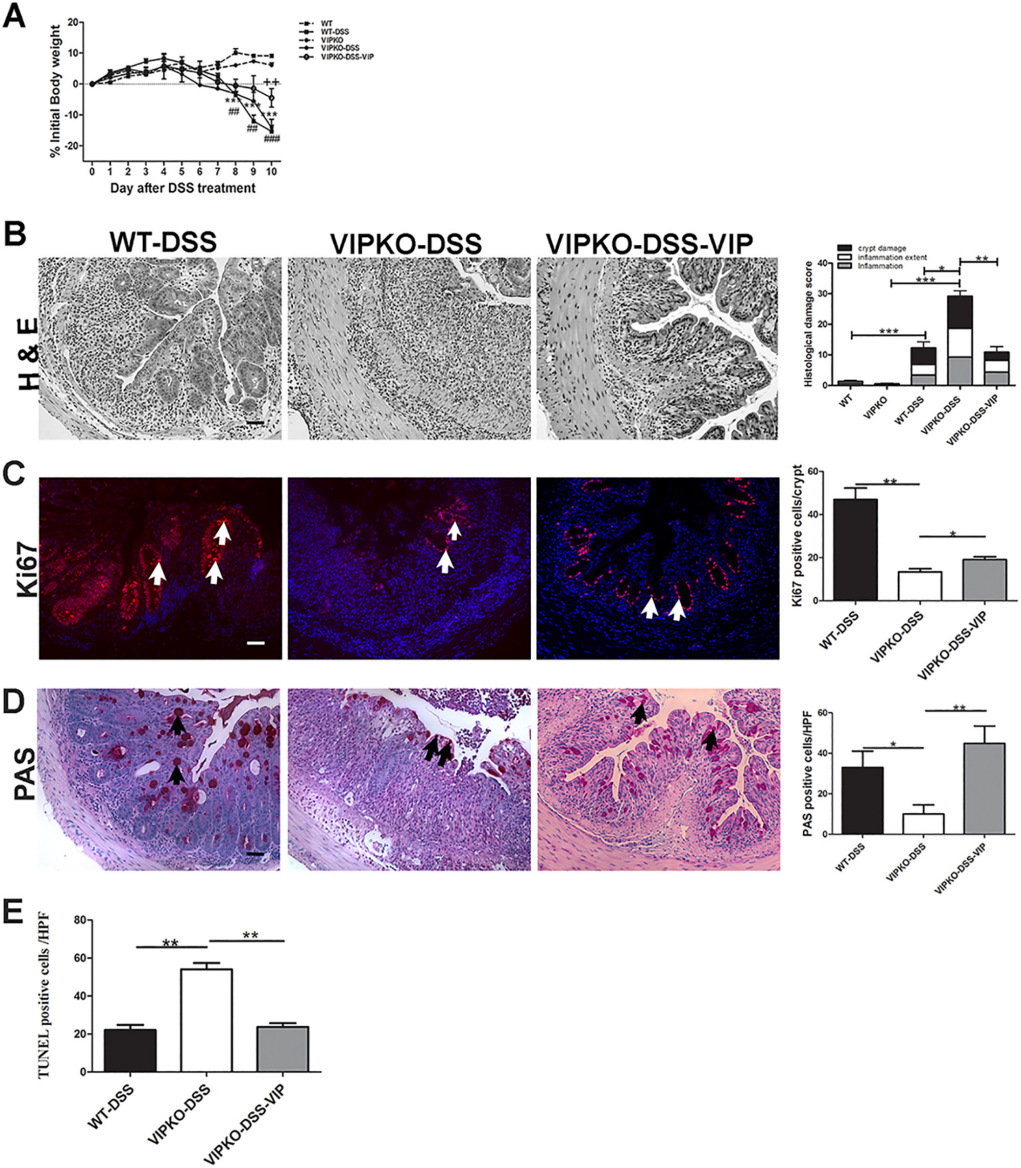
VIP protects mice against DSS-induced colitis. Exogenous VIP was administered to DSS-treated VIPKO mice daily for 10 days. Change in body weight over the 10-day study period,in naive WT and VIPKO mice, DSS exposed WT (WT-DSS), VIPKO (VIPKO-DSS) and DSS plus VIP treated VIPKO (VIPKO-DSS-VIP) mice (A). Representative H&E stained colon sections and histological damage score at day 10 in DSS exposed WT (WT-DSS), VIPKO (VIPKO-DSS) and DSS plus VIP treated VIPKO (VIPKO-DSS-VIP) mice (B), IEC proliferation determined by Ki67 immunostaining (C), selective labeling of neutral mucins with PAS and quantification of PAS+ve cells as total number of PAS+ve cells/HPF (D) and cell death determined by TUNEL staining (E); n = 6–7 animals/ group, results are represented as means ± SEM, *P<0.05, **P< 0.01, ***P<0.001. Scale bar = 50 μm. Arrows highlight Ki67 +ve cells in (C) and PAS+ve cells in (D).

To determine if the increased susceptibility of VIPKO mice at our facility to develop severe mucosal damage during chemically induced colitis was unique to DNBS, we also induced colitis in these mice using DSS. As seen with DNBS treatment, VIPKO mice developed less severe diarrhea than WT mice. DSS treated VIPKO mice showed increased disease activity, including increased weight loss at day 5–6 post-DSS, compared to WT mice (Fig 6A). At day 10, DSS treated VIPKO mice had significantly more blood detectable in their stool compared to DSS-treated WT mice (rectal score 0.9 ± 0.17 *vs*. 0.27 ± 0.09, p<0.05). Moreover, colitic VIPKO mice showed severe histological mucosal damage in the distal colon, characterized by complete crypt drop out, widespread ulceration and marked transmural infiltration of neutrophils and mononuclear inflammatory cells (Fig 6B). In contrast, tissues of colitic WT mice retained crypt structure, showing only moderate inflammatory infiltrate and microscopic colitis scores were significantly lower than DSS- VIPKO mice (12.28 ± 3.77 *vs*. 29.14 ± 4.51 (p<0.05, Fig 6B). To address the mechanisms underlying the increased susceptibility of VIPKO mice, IEC proliferation, presence/absence of goblet cells and IECdeath were determined by Ki67, PAS and TUNEL staining, respectively. DSS challenged VIPKO mice had dramatically fewer Ki67+ve IEC in the distal colon, compared to DSS-WT mice (13.39 ± 1.40 *vs*. 47.05 ± 5.22, p<0.01, Fig 6C) and had significantly more TUNEL+ve cells (presumed IEC in lumen and apex of crypts) than DSS-WT mice (47.29 ± 3.98 *vs*. 22.80 ± 1.99, p<0.01, Fig 6E), indicating altered cell death and turnover in VIPKO mice. Moreover, VIPKO mice had significantly fewer PAS+ve IEC in the distal colon, compared to DSS-WT mice (10.00 ± 4.52 *vs*. 33.00 ± 8.07, p<0.05, Fig 6D) suggesting the possibility of impaired mucus production/ secretion as a potential contributing factor.

Next, we examined whether VIP treatment ameliorated susceptibility of VIPKO mice to DSS challenge. VIPKO mice treated daily with VIP throughout DSS administration showed a similar phenotype to DSS treated WT mice (Fig 6A–6E), displaying a similar pattern of weight loss to DSS-WT mice and significantly higher body weights at day 10 post DSS than untreated DSS-VIPKO mice (Fig 6A, p<0.05). Moreover VIP treated DSS-VIPKO mice showed an overall reduction in rectal bleeding (data not shown), histological damage, inflammatory cell infiltration (Fig 6B), and lower histological damage scores than untreated DSS-VIPKO mice (9.28 ± 3.77 *vs*. 29.14 ± 4.51, p<0.01, Fig 6B). Additionally, DSS-VIPKO mice treated with VIP had significantly more Ki67 +ve cells (19.05 ± 1.31/crypt *vs*. 13.38 ± 13.90/crypt, p<0.05, Fig 6C), significantly more PAS +ve goblet cells (44.88 ± 8.53/HPF *vs*. 10.00 ± 4.52/HPF, p<0.01, Fig 6D) and significantly fewer TUNEL+ve cells (21.88 ± 1.35/HPF *vs*. 47.29 ± 3.78/HPF, p<0.01, Fig 6E) than untreated VIPKO mice, reaching TUNEL +ve cell numbers comparable with DSS-WT mice (21.88 ± 1.35 *vs*. 22.80 ± 1.99/HPF, p = 0.73).

Taken together, these data suggest that VIP promotes epithelial barrier homeostasis, integrity and function, thus reducing the severity of colitis and enhancing IEC recovery.

## Discussion

The present study explores the role of the enteric neuroendocrine system and particularly VIP in the regulation of intestinal barrier defenses. Over the past decade numerous studies have identified key pathways of innate defense and/or recognition of commensal microbes in the regulation of intestinal homeostasis, however studies involving the ENS have largely focused on regulation of the mucosal immune system. Using *in vivo* and *in vitro* model systems, we demonstrate that VIP plays an indispensible role in regulating colonic mucosal integrity and epithelial barrier homeostasis and its absence in an appropriate environmental context increases susceptibility to colitis. To date, there are conflicting reports on morphological changes of VIPergic neurons in the colons of IBD patients [3–5]. Moreover it is unclear whether the morphological and functional changes in the ENS in IBD patients are secondary to inflammation-induced injury. Hence, the VIPKO mouse provides a unique opportunity to evaluate the relationship between VIP and intestinal barrier integrity in the absence of inflammation-induced injury.

Data generated in this study expand on early indications that VIP plays a key role in protecting the colonic epithelium against bacterial pathogens [10]. Impaired crypt cellular dynamics including reduced IEC proliferation and migration as well as increased IEC apoptosis in the VIPKO mouse creates a vulnerable and leaky intestinal barrier that proved highly susceptible to both DSS and DNBS-induced colitis. Furthermore, the impaired IEC proliferative response and increased apoptosis likely contributed to impaired epithelial regenerative capacity of VIPKO mice. Similarly, in IBD patients, inflammation induced alterations in VIP+ neurons and its receptors [3–5] might contribute to disease pathogenesis through loss of VIP-mediated regulation of epithelial homeostasis. The heightened susceptibility to DSS in VIPKO mice observed by us differs from that observed by other researchers [12, 13]. At least two explanations may account for such a difference. One is that mice raised in different facilities or fed by different diets may show different microbiota composition. In fact, Ooi et al [35] have recently shown that feeding WT mice three different standard laboratory diets for 2 weeks resulted in very different responses to DSS challenge, which was shown to be related to changes in commensal bacteria. The other explanation is that different methodologies are used between studies. We switched to tap water from DSS for days 7–9, whereas the other studies either switched to tap water from DSS for days 5–10 or did not switch at all to tap water from DSS. The complexity of the VIP KO mouse model is further highlighted by the recent study of Abad et al, [14]. They showed that although VIP KO mice developed a milder clinical response to TNBS-induced colitis than WT mice, the histological scores and cytokine levels in the colon were similar between mouse strains. Moreover, splenocytes from TNBS-treated VIP KO mice exhibited an enhanced proliferative response to anti-CD3/CD28 stimulation in vitro [14].

Previous studies have provided insights into the relationship between ENS derived mediators and function of intestinal goblet cells in mucosal defense [24, 25, 36]. VIP has been shown to regulate MUC2 transcription [36] and secretion of mucin and TFF3 [25, 37]. Furthermore, studies indicate that Muc2 and Tff3 preserve mucosal integrity, and protect the epithelium from injury by noxious agents [38, 39]. Hence, the discovery that VIPKO mice possessed significantly fewer morphologically mature goblet cells, and produced less Muc2 and Tff3 than WT mice offers an additional explanation for their susceptibility to chemically induced colitis. Indeed, the goblet cell derived mucus layer coating the GI tract is considered the first line of mucosal defense, protecting the host from luminal microbes and other noxious agents through the barrier actions of MUC2 as well as the actions of bioactive molecules such as Tff3 [38, 39]. Defects in the thickness and/or function of the mucus layer can alter localization of commensal microbiota, increasing bacterial adhesion to mucosal surfaces. It can also increase intestinal permeability, and enhance susceptibility to colitis [38, 40], similar to the phenotype observed in our VIPKO mice. Moreover, studies have reported that in UC patients, goblet cell numbers are depleted, and mucin production is often reduced, leading to a thinner mucus layer, and impaired barrier integrity [41, 42]. Indeed, *Muc2* ^*-/-*^ mice lacking a mucus layer develop spontaneous colitis [43] and colorectal cancer [39] demonstrating that Muc2 production impacts intestinal physiology [44].

The impaired migration of IEC and goblet cells to crypt surfaces of VIPKO mice [22], as well as their defects in tissue repair likely result from their reduced expression of Tff3 [45]. Similarly, mice deficient in Tff3 (*Tff3* ^*-/-*^ *)* when challenged with DSS developed severe colitis, together with increased IEC apoptosis and poor epithelial regeneration. Notably, administration of recombinant TFF3 (rTFF3) to *Tff3* ^*-/-*^ mice restored intestinal epithelial restitution [22, 46]. Similarly, treating VIPKO mice with exogenous VIP protected IEC dynamics and goblet cell secretary capacity, reducing susceptibility to DSS-induced colitis.

The beneficial response of VIPKO mice to exogenous VIP further highlights that the VIPKO mouse phenotype can be rescued, and is thus not due to developmental defects. Moreover in preliminary experiments, we observed no gross differences in NOS, SP and NPY immunoreactivity in the myenteric plexus, consistent with a previous report [15]. A notable finding is that VIP can regulate Cdx2 expression, both *in vitro* and *in vivo*. Current data suggests that Cdx2 controls a number of IEC specific genes, while our data suggests that Cdx2 is likely involved in contributing to the balance between proliferation, migration and maturation of IEC, promoting an intact epithelial cell/ mucus defense barriers [27, 47]. Indeed, studies have demonstrated that Cdx2 targets cellular adhesion genes Claudin-2, E and L cadherin [48, 49], and MUC2 [50, 51] reinforcing the proposed role for Cdx2 as a key regulator of epithelial cellular dynamics and barrier integrity. Furthermore, there is evidence that Cdx2 expression is reduced in inflamed tissues of UC patients [52], although this might be secondary to the disease. Nevertheless, heterozygous *Cdx2* ^+/-^ mice are reported to suffer increased intestinal permeability and heightened susceptibility to DDS-induced colitis suggesting a causal relationship [53]. Given the strategic positioning of Cdx2 as a key regulator of numerous intestinal genes, and its linkage to preserving intestinal homeostasis and permeability, altered Cdx2 expression and activity in VIPKO mice most likely underlies their intestinal barrier vulnerability and enhanced sensitivity to chemically-induced colitis. Moreover, the current study shows a novel link between VIP and Cdx2 activation; however further studies are required to better understand the relation between VIP, Cdx2, and susceptibility to colitis.

In summary, VIP plays a crucial role in the development and maintenance of colonic epithelial and mucus barrier integrity, potentially through activation of Cdx2. VIP regulates colonic crypt cell proliferation, migration, and maturation, as well as secretion of bioactive goblet cell peptides, and promotes tissue repair and homeostasis, thereby controlling susceptibility to colitis. Further studies examining the role of the enteric neuroendocrine system in the regulation of intestinal barrier defense and mucosal immune responses may lead to a better understanding of IBD pathogenesis and to new avenues of therapeutic intervention in the management of patients with IBD.

## Supporting Information

S1 Fig Immunoreactive VIP +ve nerve fibres in the colonic lamina propria and submucosa (A) and myenteric plexus (B) of WT and VIPKO mice. Comparative photomicrographs were taken at the same exposure. VIP gene deletion was confirmed by PCR (C).
(TIF)

S2 Fig Colon tissues stained for 5-hydroxytryptophan (5-HT) and carbonic anhydrase-I (CA-1) in VIPKO and wild type (WT) mice. (TIF)

S1 Table Primers for real time PCR analysis. (DOCX)
