# Supplementary figures and images for: Vasoactive Intestinal Polypeptide Promotes Intestinal Barrier Homeostasis and Protection Against Colitis in Mice
Source: PLoS One. 2015 May 1;10(5):e0125225. doi: 10.1371/journal.pone.0125225 (PMC4416880; doi:10.1371/journal.pone.0125225)

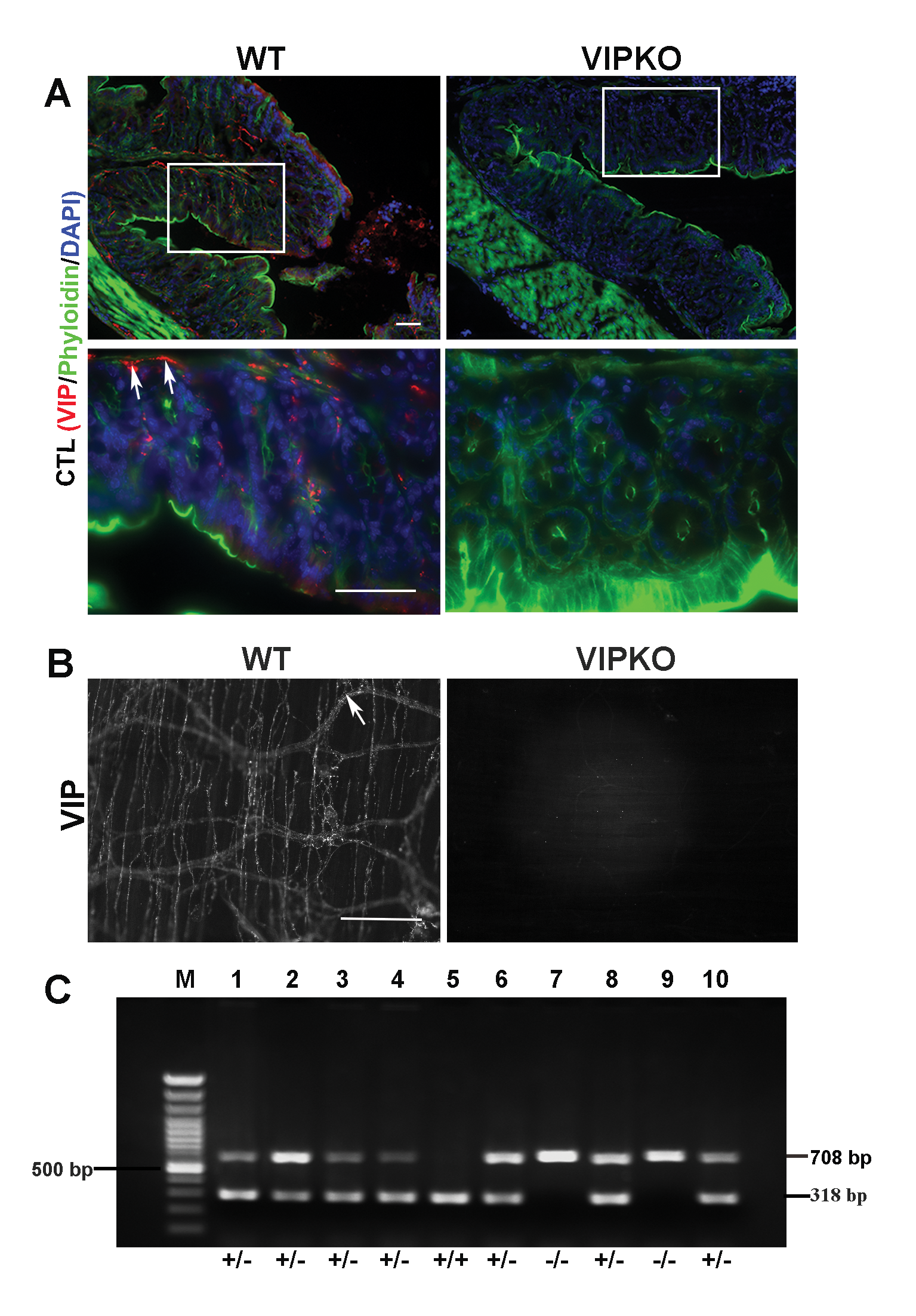

Supplement: S1 Fig — Comparative photomicrographs were taken at the same exposure. VIP gene deletion was confirmed by PCR (C). (TIF) [file pone.0125225.s001.tif]

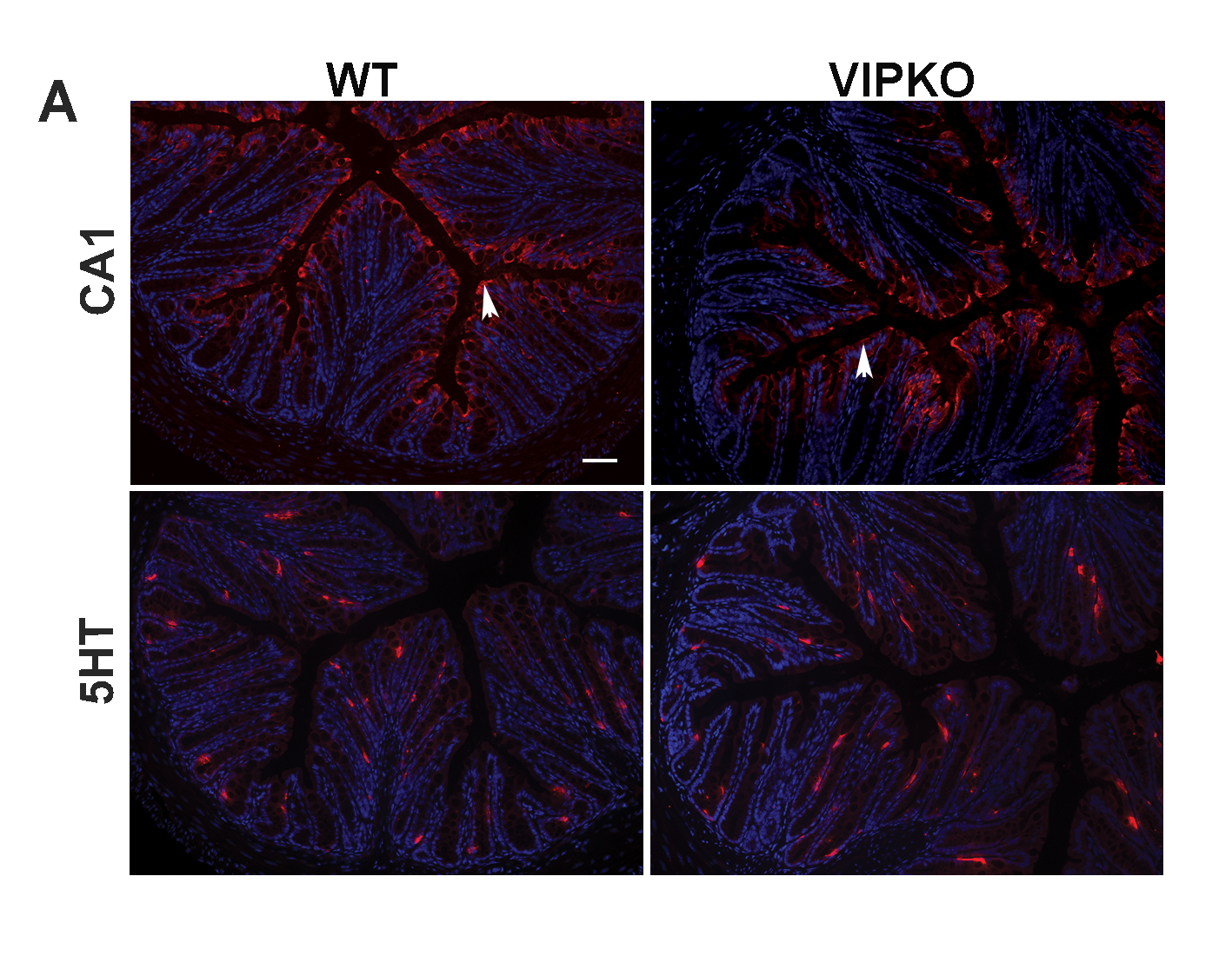

Supplement: S2 Fig — (TIF) [file pone.0125225.s002.tif]
