# Supplementary material for: Vasoactive Intestinal Polypeptide Promotes Intestinal Barrier Homeostasis and Protection Against Colitis in Mice
Source: PLoS One. 2015 May 1;10(5):e0125225. doi: 10.1371/journal.pone.0125225 (PMC4416880; doi:10.1371/journal.pone.0125225)
Supplement: S1 Table — (DOCX) [file pone.0125225.s003.docx]

Supplemental table

**S1 Table: Primers for real time PCR analysis.**

| Gene | Primer sequences | |
| --- | --- | --- |
| β-Actin | Forward:  Reverse: | 5’-CTAGGCACCAGGGTGTGAT-3’  5’-TGCCAGATCTTCTCCATGTC-3’ |
| Cdx1 | Forward:  Reverse: | 5’-AGACCGAACCAAGGACAAG-3’  5’-TGATGTACCGGCTGTAGTGAA-3’ |
| Cdx2 | Forward:  Reverse: | 5’-CGATACATCACCATCAGGAGG-3’  5’-TGGCTCTGCGGTTCTGAAA-3’ |
| KLF4 | Forward:  Reverse: | 5’-TTTCCTGCCAGACCAGATG-3’  5’-ACGACCTTCTTCCCCTCTTT-3’ |
| KFL5 | Forward:  Reverse: | 5’-CAACTCTCCCACCTGTCAGA-3’  5’-AGACGACTTTGTATAAACTTTTGTGC-3’ |
| Hes1 | Forward:  Reverse: | 5’-TCTGACCACAGAAAGTCATCA-3’  5’-AGCTATCTTTCTTAAGTGCATC-3’ |
| Math1 | Forward:  Reverse: | 5’-ATGTCCCGCCTGCTGCATGC-3’  5’-GGCGCGTGGGTCGGTGC-3’ |
| MUC2 | Forward:  Reverse: | 5’-CCCAGAAGGGACTGTGTATG-3’  5’-TGCAGACACACTGCTCACA-3’ |
| Notch1 | Forward:  Reverse: | 5’-GCTGGACAGAACTGTGAAGA-3’  5’-TCTGTACAGTACTGACCCGT-3’ |
| RELMβ | Forward:  Reverse: | 5’-GGGATGGTTGTCACTGGATGT-3’  5’-CACTGGCAGTGGCAAGTATTTC-3’ |
| TFF3 | Forward:  Reverse: | 5’-CAGATTACGTTGGCCTGTCTCC-3’  5’-ATGCTTGCTACCCTTGGCCAC-3’ |
| VPAC1 | Forward:  Reverse: | 5’-CGAAACTACATCCACATGCATCTC-3’  5’-CCATATCCTTGATGA-AGACGG-3’ |
| VPAC2 | Forward:  Reverse: | 5’-CGGTGTCTGGGACAACATCA-3’  5’-GCACTGTGACAGTTTCCCCAA-3’ |
| Wif1 | Forward:  Reverse: | 5’-AGTGTAAGTGCCCGAAAGGTTA-3’  5’-CCCTTCTATCCTCAGCCTTTTT-3’ |

|  |
| --- |

|  |
| --- |

|  |
| --- |
